# An old friend with a new face: YB-1 and its role in healthy pregnancy and pregnancy-associated complications

**DOI:** 10.3389/fcell.2022.1039206

**Published:** 2022-10-18

**Authors:** Florence Fischer, Anne Schumacher, Nicole Meyer, Beate Fink, Mario Bauer, Violeta Stojanovska, Ana Claudia Zenclussen

**Affiliations:** ^1^ Department of Environmental Immunology, Helmholtz Centre for Environmental Research, Leipzig, Germany; ^2^ Experimental Obstetrics and Gynecology, Medical Faculty, Otto-von-Guericke University, Magdeburg, Germany

**Keywords:** YB-1 protein, cold shock protein, pregnancy, implantation, trophoblast

## Abstract

By promoting tissue invasion, cell growth and angiogenesis, the Y-box binding protein (YB-1) became famous as multifunctional oncoprotein. However, this designation is telling only part of the story. There is one particular time in life when actual tumorigenic-like processes become undoubtedly welcome, namely pregnancy. It seems therefore reasonable that YB-1 plays also a crucial role in reproduction, and yet this biological aspect of the cold-shock protein has been overlooked for many years. To overcome this limitation, we would like to propose a new perspective on YB-1 and emphasize its pivotal functions in healthy pregnancy and pregnancy-related complications. Moreover, we will discuss findings obtained from cancer research in the light of reproductive events to elucidate the importance of YB-1 at the feto-maternal interface.

## Graphical Abstract

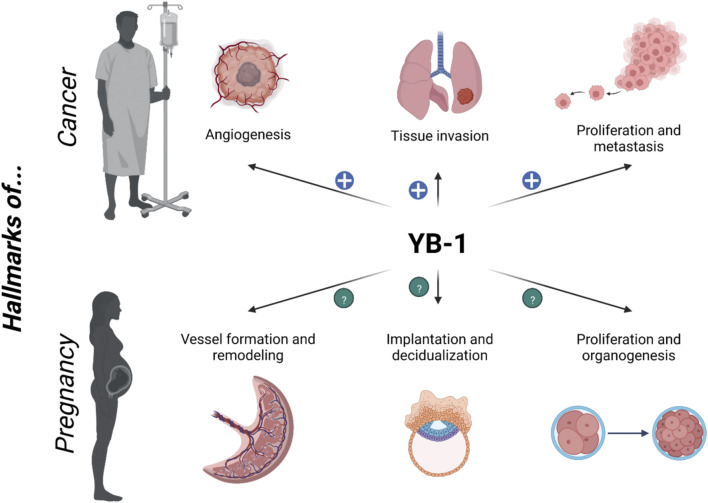

## Introduction

As hallmarks of cancer, tissue invasion, cell growth and angiogenesis are terms that scare physicians and scientists. However, there is a time in every individuals’ life when these processes appear in another light, namely pregnancy. From conception on, the fertilized oocyte—the zygote—experiences remarkable transformations that finally result in the birth of a child. The uterus has to adapt to the needs of the fetus and also undergoes a multitude of tissue remodeling processes including decidualization and spiral artery (SA) conversion. Finally, the placentation which involves angiogenesis and tissue invasion events ensures the appropriate nutrient and oxygen supply to the developing baby.

The Y-box binding protein 1 (YB-1) made itself a name as a multifunctional DNA- and RNA-binding protein that regulates many cellular functions including cell proliferation, migration, invasion and stress response. Briefly, after the discovery of YB-1, it was found to be overexpressed in several tumors. Consequently, the research on YB-1 has focused mainly on its role as an oncoprotein and its use as prognostic marker and therapeutic target in cancer (Yin et al., 2022). Moreover, several studies investigated the association between YB-1 and inflammation (Rybalkina and Moiseeva, 2022). However, there is one aspect that has been missed in the research on YB-1 for almost 30 years, namely its functions in pregnancy at the feto-maternal interface. In this article, we aim to provide a new perspective on YB-1 and highlight its role in reproduction and early development.

## YB-1 at a glance

Y-box binding (YB) proteins belong to the evolutionary highly conserved family of cold shock proteins (Lindquist and Mertens, 2018). These proteins get their name from the existence of one or more nucleic acid binding cold shock domains (CSD) which endow the proteins with a multitude of functions related to transcription, translation and mRNA splicing (Lindquist and Mertens, 2018). In humans and mice, the YB family consists of three members: YB-1, -2- and -3, with YB-1 being the best characterized protein.

YB-1 was discovered in 1988 as a negative regulator of transcription of the HLA-DR β chain gene (Didier et al., 1988). It can bind to DNA sequences called Y-boxes (5′-CTGATTGG -3′) which are located in the promoter region of the target gene (Didier et al., 1988). Besides being a transcription factor, YB-1 can also stimulate or inhibit translation, for instance by regulating mRNA packaging and stability [reviewed in (Mordovkina et al., 2020)]. Moreover, YB-1 can be secreted and serve as auto- and paracrine factor that regulates proliferation and migration (Frye et al., 2009). Because YB-1 is remarkably multifunctional and orchestrates many cellular functions that basically determine the fate of a cell, its activity needs to be tightly controlled. The functional fine tuning of YB-1 is achieved by several post-translational modifications including phosphorylation, methylation, acetylation and ubiquitylation (Yin et al., 2022).

After its discovery, YB-1 quite quickly became famous as an oncoprotein. Associations were found for YB-1 and both solid and hematopoietic cancer such as breast cancer (Bargou et al., 1997), osteosarcoma (Oda et al., 1998), colorectal carcinoma (Shibao et al., 1999), ovarian serous adenocarcinoma (Kamura et al., 1999), lung cancer (Shibahara et al., 2001) and refractory acute B-cell leukemia (Kariminia et al., 2017). However, mutations of the *YBX1* gene seem to be rather rarely the case and can be found in only around 1% of all cancer types (Johnson et al., 2019). Instead, tumor cells often tend to overexpress this protein and/or favor its nuclear translocation. Especially the latter is associated with a poor prognosis and a highly aggressive course of disease (Kamura et al., 1999). The oncogenic characteristics of YB-1 have already been comprehensively reviewed (Lasham et al., 2013; Johnson et al., 2019; Shah et al., 2021; Yin et al., 2022) and here will be summarized only in short.

YB-1 can intervene in several carcinogenic processes such as proliferation, stemness, migration and metastasis, invasion, immune escape and multidrug resistance (Johnson et al., 2019). Following the discovery of the HLA-DR β chain gene as the first gene whose expression is regulated by YB-1 (Didier et al., 1988), more and more studies demonstrated that the cold shock protein promotes the transcription of genes involved in several carcinogenic pathways. For instance, the nuclear accumulation of YB-1 leads to increased expression of cyclin A and B1 that favor cell cycle progression (Jurchott et al., 2003) and of cell growth promoting genes such like epidermal growth receptor (Stratford et al., 2007). Beside transcriptional effects, YB-1 can also promote cell survival *via* its function as translation factor. For instance, a mutation in the untranslated region of the *c-myc* gene, that has been described in multiple myeloma, favors a strong binding of YB-1 and thus increased the synthesis of the protein (Cobbold et al., 2010). Finally, some tumors also actively secrete YB-1 and make use of it as an intracellular communication factor. Kosnopfel and colleagues have shown that YB-1 secreted by melanoma cells stimulates tumor cell migration and invasion (Kosnopfel et al., 2020). Although promising results from *in vitro* and *in vivo* models, to date, there is no established therapy that specifically targets YB-1 in clinical practice (Shah et al., 2021).

## YB-1 in pregnancy and related disorders

After an oocyte becomes fertilized and reaches the uterine cavity, trophoblast cells of the blastocyst attach to the epithelial layer of the endometrium, the so-called decidua. In the following process of placentation, embryonic cells invade the maternal tissue in order to establish a vascular infrastructure that supports the developing fetus with oxygen and nutrients. The remodeling of uterine SA from vessels with a low diameter into arteries with a vein-like structure including thin walls and high diameters, as well as the formation of new vessels, are also vital for an adequate blood perfusion of the placenta. The physiological condition of pregnancy sheds a very different light on tissue invasion, cell growth and angiogenesis. Certain molecular pathways that are devastating in terms of carcinogenesis are essential for successful pregnancy. While YB-1 was intensively studied in different types of cancer, our knowledge about its role in pregnancy is very limited.

Among the YB proteins, YB-1 seems to be developmentally most important: In comparison to the loss of YB-2 and YB-3, YB-1 is mostly embryonic lethal as demonstrated in YB-1 null mutant (YB1^−/−^) mice (Lu et al., 2005; Uchiumi et al., 2006). However, the serious consequences of YB-1 deficiency become only evident after the first trimester, when a functional placenta is already established (Meyer et al., 2020). This suggests that implantation and early decidualization are not dependent on YB-1 or that there are efficient compensatory mechanisms. However, since the effect of YB-1 on early gestational events has not been investigated yet in detail, this remains speculative.

After placentation which finishes around gestation days (GD) 14–15 in mice, null mutation and heterozygous YB-1 knockout mice suffer from IUGR (Lu et al., 2005; Uchiumi et al., 2006; Meyer et al., 2020). Despite a small proportion of YB1^−/−^ mice is born alive, most of them have severe craniofacial defects, multi-organ hypoplasia and do not survive the first day of life (Lu et al., 2005; Uchiumi et al., 2006). The multi-organ hypoplasia might be a consequence of reduced cell proliferation (Lu et al., 2005).

In addition to the fetal growth impairment, also the placenta showed certain functional and structural alterations in YB-1 deficiency. We have been able to show that IUGR might be a result of placenta insufficiency demonstrated by increased placental diameter/thickness ratio and weight at GD14 as well as inadequate SA remodeling in trophoblast-specific YB-1 deficient mice (Meyer et al., 2020). By overexpressing or downregulating YB-1 in two different trophoblast cell lines HTR8/SVneo and JEG3, we aimed to disclose the cellular mechanism underlying the placental dysfunction (Stojanovska et al., 2021). According to the aforementioned findings, we showed that overexpression of YB-1 increased proliferation, whereas its knockdown had anti-proliferative effects in the trophoblast cell lines (Stojanovska et al., 2021). Moreover, genes mediating migration, invasion, apoptosis, and inflammation were altered by YB-1 downregulation (Stojanovska et al., 2021). Especially the secretion of the cytokine IL-6, which is involved in trophoblast differentiation, proliferation and migration (Goyal et al., 2013), was remarkably enhanced in HTR8/SVneo cells. Altogether, these studies provide evidence for a crucial role of YB-1 in placentation and fetal development.

Several studies also investigated the expression and biological activity of YB-1 in the uterus, interestingly, primarily in the context of endometriosis. Endometriosis is an inflammatory disease in which tissue similar to the uterine lining grows outside of the uterine cavity, often at the external uterine wall, uterine tubes and at the ovaries. Elevated serum YB-1 concentrations and an increased expression of YB-1 in endometrial tissue, especially in uterine epithelial cells, was found in patients suffering from endometriosis in comparison to control patients without endometriosis (Silveira et al., 2012; Ahrens et al., 2015). In addition, several *in vitro* and *in vivo* interventions that aim to suppress YB-1 yielded mechanistical insight. In an endometrial cell line, YB-1 inhibition resulted in reduced proliferation, increased cellular apoptosis rates and tendentially a decreased invasive potential of the cells (Silveira et al., 2012; Silveira et al., 2017). Furthermore, the pharmacological inhibition of YB-1 suppressed the growth of peritoneal endometria implants in mice (Silveira et al., 2017). These studies indicate that in the pathophysiological condition of endometriosis, YB-1 seems to promote cell proliferation, survival and invasion. Whether YB-1 also orchestrates the physiological uterine tissue remodeling during the estrous cycle and pregnancy is still unknown.

Especially the knowledge about the physiological importance of YB-1 in human decidualization and placentation is very limited. Only recently, we investigated the expression of YB-1 in human pregnancy and different pregnancy-related complications: While YB-1 gene expression was upregulated in the placenta of women with preeclampsia, IUGR was associated with lower YB-1 expression in comparison to term pregnancies (Stojanovska et al., 2021). The latter was additionally confirmed by measurement of YB-1 in the serum of the mother (Stojanovska et al., 2021). These results indicate for the first time that both an increased and decreased YB-1 expression can lead to diverse pregnancy-related complications (Stojanovska et al., 2021). We are only beginning to reconsider YB-1 not only as an oncoprotein but also as a key player in pregnancy.

## Possible functions of YB-1 at the feto-maternal interface

Proliferation, invasion and angiogenesis are essential for the beginning and course of pregnancy. Notably, YB-1 was shown to be involved in all these processes. However, this knowledge is mainly derived from research on cancer, which shares some physiological characteristics with pregnancy. These parallels rise inevitably a question: what can we learn from cancer about the role of YB-1 in pregnancy? In the following, we will discuss a selection of potential mechanisms that are known to be regulated by YB-1 and that might be crucial for pregnancy. Additionally, we performed transcriptional analysis of uterine/decidual tissue isolated from pregnant (GD14) heterozygous YB-1 mice in comparison to wildtype mice to proof our assumptions.

Tissue invasion and angiogenesis require the breakdown of physical barriers. A class of zinc-dependent endopeptidases, so-called matrix metalloproteinases (MMPs), facilitate these tissue remodelling processes by their ability to degrade extracellular matrix proteins such as fibronectin and collagen (Wen et al., 2020). Thereby, they became an interesting aspect of tumor biology and indeed MMPs are involved in all stages of carcinogenesis from proliferation to invasion and metastasis (Kessenbrock et al., 2010). Interestingly, YB-1 regulates several matrix metalloproteinases including MMP-1 (Lim et al., 2019), MMP-2 (Mertens et al., 1999; Matsumoto et al., 2005), MMP-9 (Stojanovska et al., 2021), MMP-11 (Li et al., 2017), MMP-12 (Samuel et al., 2005), MMP-13 (Samuel et al., 2007), MMP-14 (Lovett et al., 2010) and MMP-15 (Shinkai et al., 2016). It does not only act as transcription and translation factor, but also promote the cellular turnover and recycling rates of MMPs and thereby modulate the invasive and metastatic potential of cancer cells (Lovett et al., 2010; Lim et al., 2019).

There is only very limited data about the regulatory effect of YB-1 on MMPs at the feto-maternal interface, yet these enzymes fulfil several important functions during pregnancy. For instance, MMP-2 and MMP-9 are expressed in human extra-villous trophoblasts respectively villous cytotrophoblasts (Isaka et al., 2003). Both enzymes are directly related to the trophoblast invasiveness and migratory potential into the maternal decidua (Su et al., 2017). Especially MMP-9 may also contribute to SA remodeling and placental neovascularization as it was shown to promote angiogenesis in prostate cancer (Bruni-Cardoso et al., 2010). From cancer studies, we already know that YB-1 can modulate MMP-2, however, dependent on the tissue localization it can have both enhancing and suppressive effects: While it induced the expression of MMP-2 in human melanoma and hepatocellular carcinoma cells (Sechi et al., 2018; Liao et al., 2020), YB-1 had repressive effects on this enzyme in human HeLa cervical carcinoma cells (Samuel et al., 2005; Samuel et al., 2007). A similar phenomenon was recently shown by us: We found that YB-1 silencing modulated the expression of MMP-2 and MMP-9 in human trophoblasts (Stojanovska et al., 2021). However, the direction of the effect was dependent on both the specific cell line and the shRNA construct that was used for repression of YB-1 (Stojanovska et al., 2021). From these results, we can assume that the expression and/or activity of several MMPs at the feto-maternal interface is likely dependent on YB-1. Still, supporting data about the relevance of YB-1 for MMP expression at different fetal and maternal compartments *in vivo* is largely lacking.

**FIGURE 1:**
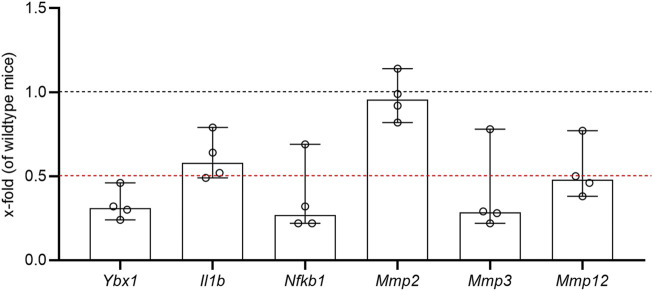
Transcriptional analysis of genes related to pregnancy. The expression of indicated genes was measured by RT-PCR in uterine/decidual tissue of heterozygous YB-1 and wildtype mice at GD14. The graphic shows the x-fold-expression of the heterozygous YB-1 mice in comparison to the wildtype group according to ddCt method. *n* = 4 (for method details and primer sequences see supplementary material). *Ybx1*, Y-box binding protein 1β; *Il1b*, interleukin beta; *Nfkb1*, nuclear factor kappa-light-chain-enhancer of activated B cells subunit 1; *Mmp2/3/12*; matrix metallopeptidase 2/3/12.

In order to contribute to close this gap of knowledge, we performed gene transcription analysis of several MMPs in the uterine/decidual tissue isolated from pregnant (GD14) heterozygous YB-1 mice in comparison to wildtype mice. While we found no effect on the expression of MMP-2, YB-1 deficiency resulted in the repression of MMP-3 and MMP-12 at GD14 (Figure 1). To the best of our knowledge, this is the first report of a regulatory relationship between YB-1 and MMP-3, also called stromelysin-1. MMP-3 is expressed in trophoblasts, especially during first trimester of human pregnancy (Husslein et al., 2009), and in baboon endometrial stromal fibroblasts (Strakova et al., 2003). In the murine uterus it was also shown to be expressed during early and mid-time gestation (Lombardi et al., 2018). MMP-3 seems to be crucial for decidualization (Strakova et al., 2003) and SA remodeling (Pan et al., 2022). Moreover, MMP-3 activates MMP-9, which is critical for angiogenesis as mentioned before (Tu et al., 2021). Several polymorphisms in the gene or a low expression of MMP-3 were reported in recurrent pregnancy loss (Balci and Özdemir, 2019; Behforouz et al., 2021), while an increased expression was associated with early-onset preeclampsia and preterm birth (Sundrani et al., 2013; Laskowska, 2017). Another MMP that turned out to be possibly regulated by YB-1 in the uterine/decidual tissue was the membrane-bound macrophage metalloelastase MMP-12. This enzyme is not only expressed in macrophages as the name suggests, but also in first trimester trophoblasts, decidual stroma and endothelial cells (Harris et al., 2010). In humans, a high expression of MMP-12 was observed in the first trimester placenta, while the expression decreased until end of first trimester and diminished in term placenta (Hiden et al., 2018). MMP-12 plays a crucial role in the uterine SA remodeling and its downregulation is associated with pregnancy-related disorders such as fetal growth restriction and pre-eclampsia (Lian et al., 2010). On the other side, higher placental expression of MMP-12 was seen to be associated with preeclampsia (Zhao et al., 2021). The literature already indicates that YB-1 regulates MMP-12, however, contrasting to our results, it was reported to have rather suppressive effects on the enzyme in cervical cancer cells (Samuel et al., 2005; Lin et al., 2021).

In summary, the regulatory effect of YB-1 on MMPs is a representative example of its potential roles in reproduction. Nevertheless, there are still several limitations of our understanding. For example, since the direction of YB-1’s effects seems to be highly dependent on the cellular environment, further research is needed to disclose the expression of YB-1 and MMPs at different feto-maternal compartments during the course of pregnancy. Moreover, we cannot conclude from the present point of view whether YB-1 directly influences the expression of MMPs or rather modulates upstream targets. For instance, several cytokines, including IL-1β and TNF, and the transcription factor NFκB—some of which were indeed repressed in uterine/decidual tissue of heterozygous YB-1 mice (Figure 1)—were also shown to control the expression of MMPs and thus may mediate the effects of YB-1 (Husslein et al., 2009; Souslova et al., 2010; Sanchavanakit et al., 2015). Therefore, interventional studies using YB-1 knockdown or overexpressing approaches are needed to close this gap of knowledge and to elucidate the actual molecular pathways and cellular mechanisms underlying YB-1’s impact on MMPs and potential targets beyond (see Supplementary Figure S1).

## Conclusion and future directions

The multifunctionality of YB-1 is known for almost 30 years, research on this cold shock protein was, however, mainly focused on its role in cancer. Therefore, we are only at the beginning to consider YB-1 as a regulator of processes that contribute to successful pregnancy. Yet, YB-1 deficiency or overexpression seems to disturb physiological pregnancy which let the protein to become an interesting diagnostic and/or therapeutic marker for several obstetric complications. Mechanistical studies on YB-1 in different types of cancer can guide future research and hint at promising targets of YB-1. Nevertheless, the functionality of YB-1 is complex and often dependent on the tissue environment. There is a great need for studies that provide knowledge about the expression of YB-1 at the feto-maternal interface during different stages of healthy pregnancy and in different pregnancy-related complications. Moreover, the disclosure of potential downstream targets would lead to both the better understanding of important reproductive processes such as decidualization and placentation and the suitability of YB-1 for therapeutic interventions. (Sahay et al., 2018).

## Methods

### Animal maintenance

Male and female C57BL/6 wild-type (WT, thus YB-1^+/+^) and heterozygous (HET, YB-1^+/−^) YB-1 mice were bred and maintained at the animal facility of the Magdeburg University. All mice were kept in a 12 h light/dark cycle at 22 ± 2°C and an air humidity of 40%–60%. Mice received water and food ad libitum.

### Experimental design and sample collection

Animal experiments were performed according to the institutional guidelines upon ministerial approval (Landesverwaltungsamt Sachsen-Anhalt: 42502-2-1327 Uni MD). All experiments were conducted by authorized persons according to the Guide for Care and Use of Animals in Agriculture Research and Teaching. Eight- to eleven-week-old YB-1^+/−^ females were mated with YB-1^+/−^ males. YB-1^+/+^ wildtype females paired with YB-1^+/+^ wildtype males were used as a control. Female mice were checked twice a day for the appearance of a vaginal plug that indicated gestation day (GD) 0 of gestation. Animals were sacrificed at GD 14 and the uterine/decidual tissue was isolated and stored at −80°C.

### RNA isolation, cDNA synthesis and RT-PCR

After addition of 1 ml Trizol Reagent (Thermofisher Sientific, MA, USA), the uterine/decidual tissue was dissociated by using a Tissue Lyser LT (6 min, 50 1/s, 2 stainless steel beads per vial; Qiagen, Hilden, Germany). Total RNA was isolated according to manufacturer’s instruction. 1000 ng RNA were used for cDNA synthesis with the RevertAid™ H Minus Reverse Transcriptase kit (Thermo Fisher Scientific, MA, USA). RT-PCR was performed with SYBRgreen I nucleic acid gel stain (Thermo Fisher Scientific, MA, USA) on a LightCycler 480 (Roche Applied Sciences, Penzberg, Deutschland) with the following cycling conditions: 5 min at 95°C, followed by 45 cycles of 95°C for 20 s, 1 min at 60°C and 72°C for 30 s or with Universal Probe Library probes using the BioMark™ HD System (Fluidigm). All reactions were run in triplicates. Sequences of exon-spanning primers of target genes are listed in Supplementary Table S1. To quantify the relative expression, the expression of the gene of interest was normalized to the reference gene beta-actin (actb; dCt) and to the control condition (wildtype mice, ddCt).

### Graphical Abstract

The graphical abstract was created with BioRender.com.

## Data Availability

The raw data supporting the conclusion of this article will be made available by the authors upon request without undue reservation.

## Supplementary material

SUPPLEMENTARY FIGURE S1 Transcriptional analysis of genes related to proliferation, immune response, angiogenesis and invasion in YB-1 deficient mice. The expression of indicated genes was measured by RT-PCR in uterine/decidual tissue of heterozygous YB-1 and wildtype mice at GD14. Shown is the fold-expression of the heterozygous YB-1 mice in comparison to the wildtype group according to ddCt method. *n* = 4. *Egfr*, epidermal growth factor receptor; *Notch1*, neurogenic locus notch homolog protein 1; *Zeb1*, zinc finger E-box binding homeobox 1; *Fasl*, fas ligand; *Stat3*, signal transducer and activator of transcription 3; *Tnfrsf1b*, tumor necrosis factor receptor superfamily member 1b; *Tgfb1*, transforming growth factor beta 1; *Cxcl1/5*, C-X-C motif chemokine ligand 1/5; *Ccl2/11*, C-C motif chemokine ligand 2/11; *Csf3*, colony stimulating factor 3; *Timp1/2/3*, tissue inhibitor of metalloproteinase 1/2/3; *Mtor*, mechanistic target of rapamycin kinase; *Hif1a*, hypoxia inducible factor 1 subunit alpha; *Ang*, angiogenin, *Serpine1*, serine (or cysteine) peptidase inhibitor, clade E, member 1; *Snai1*, snail family zinc finger 1.

SUPPLEMENTARY TABLE S1 List of primers used in the present study. UPL, Universal Probe Library probe.
