# Supplementary material for: An old friend with a new face: YB-1 and its role in healthy pregnancy and pregnancy-associated complications
Source: Front Cell Dev Biol. 2022 Oct 18;10:1039206. doi: 10.3389/fcell.2022.1039206 (PMC9624282; doi:10.3389/fcell.2022.1039206)
Supplement: Supplementary file 1 [file DataSheet1.pdf]

Supplementary Material

An old friend with a new face: YB-1 and its role in healthy pregnancy and pregnancy-associated complications

Florence Fischer[1], *Anne Schumacher*[1,2], *Nicole Meyer*[1,2], *Beate Fink*[1], *Mario Bauer*[1], *Violeta Stojanovska*[1,2], *Ana Claudia Zenclussen*[1,2]

[1] Department of Environmental Immunology, Helmholtz Centre for Environmental Research, Leipzig, Germany

[2] Experimental Obstetrics and Gynecology, Medical Faculty, Otto-von-Guericke University, Magdeburg, Germany

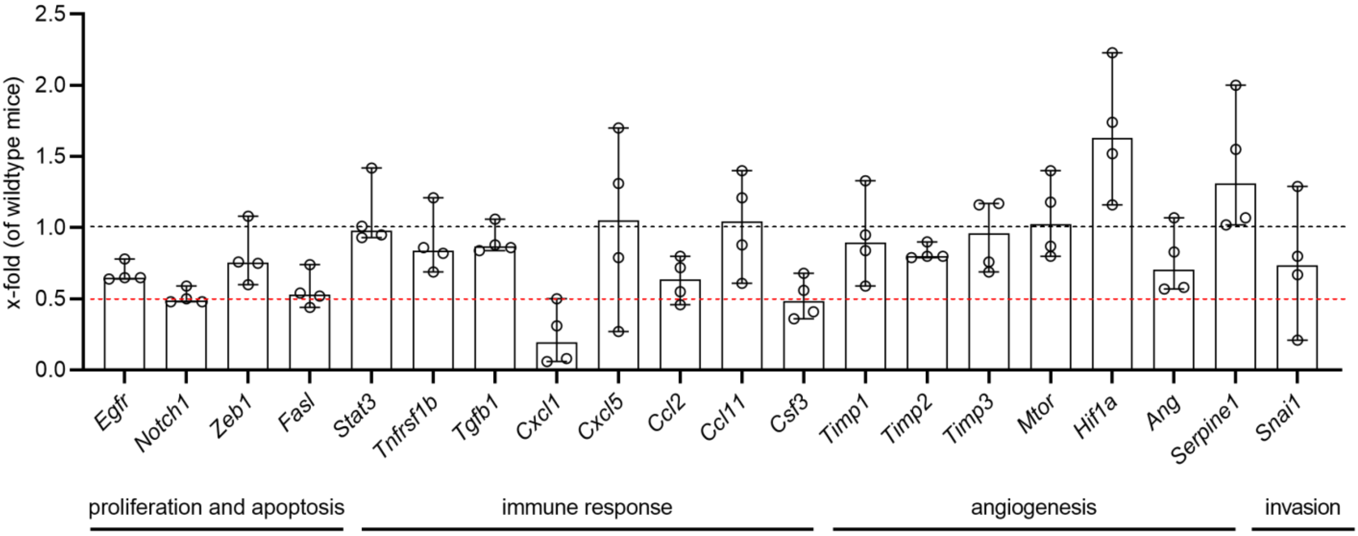

Supplementary Figure 1. Transcriptional analysis of genes related to proliferation, immune response, angiogenesis and invasion in YB-1 deficient mice. The expression of indicated genes was measured by RT-PCR in uterine/decidual tissue of heterozygous YB-1 and wildtype mice at GD14. Shown is the fold-expression of the heterozygous YB-1 mice in comparison to the wildtype group according to ddCt method. n = 4. *Egfr*, epidermal growth factor receptor; *Notch1*, neurogenic locus notch homolog protein 1; *Zeb1*, zinc finger E-box binding homeobox 1; *Fasl*, fas ligand; *Stat3*, signal transducer and activator of transcription 3; *Tnfrsf1b*, tumor necrosis factor receptor superfamily member 1b; *Tgfb1*, transforming growth factor beta 1; *Cxcl1/5*, C-X-C motif chemokine ligand 1/5; *Ccl2/11*, C-C motif chemokine ligand 2/11; *Csf3*, colony stimulating factor 3; *Timp1/2/3*, tissue inhibitor of metalloproteinase 1/2/3; *Mtor*, mechanistic target of rapamycin kinase; *Hif1a*, hypoxia inducible factor 1 subunit alpha; *Ang*, angiogenin, *Serpine1*, serine (or cysteine) peptidase inhibitor, clade E, member 1; *Snai1*, snail family zinc finger 1.

Supplementary Table 1. List of primers used in the present study. UPL, Universal Probe Library probe

Gene	Forward primer	Reverse Primer	UPL
Actb	aaggccaaccgtgaaaagat	gtggtacgaccagaggcatac	56
Ang	aacctcaccctgcaaagatg	agtggacaggcaaaccattc	85
Ccl11	agagctccacagcgcttct	gcaggaagttgggatgga	18
Ccl2	gtccctgtcatgcttctgg	cgttaactgcatctggctga	19
Csf3	gagcagttgtgtgccaccta	cgatagagcctgcaggagac	84
Cxcl1	gactccagccacactccaac	tgacagcgcagctcattg	83
Cxcl5	ttcttgggtgtgttaagagtgttc	tctgcatgacacagcagctt	26
Egfr	gaccttcacatcctgccagt	cgccaaagaaaactgaccat	63
Fasl	accggtggtatttttcatgg	tttaaggctttggttggtgaa	21
Gapd	gggttcctataaatacggactgc	ccattttgtctacgggacga	52
Hif1a	gctgaagacacagaggcaaa	tcatcagtggtggcagttgt	59
Il1b	agttgacggaccccaaaag	tttgaagctggatgctctcat	26
Mmp12	ccacttcgccaaaaggttta	ggggtaagcagggtccat	51
Mmp2	aactttgagaaggatggcaagt	tgccacccatggtaaacaa	29
Mmp3	ttgttctttgatgcagtcagc	gatttgcgccaaaagtgc	7
Mtor	ctcaaggcctgatgggatt	gttcatggtgtcctggaggt	31
Nfkb1	agcttcactcggagactgga	aactccgccattttcttcct	52
Notch1	cctcagcacaccgtgtaaga	tcagcctgctgacatgattt	13
Rplp0	ctgctgaacatgctgaacatc	tgtcgagcacttcagggtta	62
Serpine1	aggatcgaggtaaacgagagc	gcgggctgagatgacaaa	69
Snai1	cggtgaccccgactaccta	ggggtaccaggagagagtcc	12
Stat3	cgatgcctgtgggaagag	gtcactacggcggctgtt	25
Tgfb1	gcaacatgtggaactctaccag	cagccactcaggcgtatca	66
Timp1	gcaaagagctttctcaaagacc	agggatagataaacagggaaacact	76
Timp2	ttttgcaatgcagacgtagtg	ggaatccacctccttctcg	21
Timp3	cacggaagcctctgaaagtc	tcccacctctccacaaagtt	62
Tnfrsf1b	agtgcatgaggctgagcaa	acatttccatttggggctct	81
Ubc	gtctgctgtgtgaggactgc	cctccagggtgatggtctta	77
Ybx1	acgcccagagaaccctaaac	gtcagagggcaaaaagcaag	
Zeb1	ggaaaccgcaagttcaagtg	ctgatctaggcctgccattc	36
